# Impact of Blood Culture Contamination on Antibiotic Use, Resource Utilization, and Clinical Outcomes: A Retrospective Cohort Study in Dutch and US Hospitals

**DOI:** 10.1093/ofid/ofad644

**Published:** 2023-12-22

**Authors:** Michiel Schinkel, Anneroos Boerman, Karen Carroll, Sara E Cosgrove, Yea-Jen Hsu, Eili Klein, Prabath Nanayakkara, Rogier Schade, W Joost Wiersinga, Valeria Fabre

**Affiliations:** Center for Experimental and Molecular Medicine, Amsterdam UMC, Location Academic Medical Center, Amsterdam, the Netherlands; Division of Acute Medicine, Department of Internal Medicine, Amsterdam UMC, Location Vrije Universiteit Amsterdam, Amsterdam, the Netherlands; Division of Acute Medicine, Department of Internal Medicine, Amsterdam UMC, Location Vrije Universiteit Amsterdam, Amsterdam, the Netherlands; Department of Pathology, Johns Hopkins University School of Medicine, Baltimore, Maryland, USA; Division of Infectious Diseases, Department of Medicine, Johns Hopkins University School of Medicine, Baltimore, Maryland, USA; Department of Health Policy and Management, Johns Hopkins Bloomberg of School of Public Health, Baltimore, Maryland, USA; Department of Emergency Medicine, Johns Hopkins University School of Medicine, Baltimore, Maryland, USA; Center for Disease Dynamics, Economics & Policy, Washington, DC, USA; Division of Acute Medicine, Department of Internal Medicine, Amsterdam UMC, Location Vrije Universiteit Amsterdam, Amsterdam, the Netherlands; Department of Medical Microbiology and Infection Prevention, Amsterdam UMC, Amsterdam, the Netherlands; Center for Experimental and Molecular Medicine, Amsterdam UMC, Location Academic Medical Center, Amsterdam, the Netherlands; Division of Infectious Diseases, Department of Internal Medicine, Amsterdam UMC, Location Academic Medical Center, Amsterdam, the Netherlands; Division of Infectious Diseases, Department of Medicine, Johns Hopkins University School of Medicine, Baltimore, Maryland, USA

**Keywords:** antibiotic stewardship, blood culture contamination, blood culture quality

## Abstract

**Background:**

Blood culture contamination (BCC) has been associated with prolonged antibiotic use (AU) and increased health care utilization; however, this has not been widely reevaluated in the era of increased attention to antibiotic stewardship. We evaluated the impact of BCC on AU, resource utilization, and length of stay in Dutch and US patients.

**Methods:**

This retrospective observational study examined adults admitted to 2 hospitals in the Netherlands and 5 hospitals in the United States undergoing ≥2 blood culture (BC) sets. Exclusion criteria included neutropenia, no hospital admission, or death within 48 hours of hospitalization. The impact of BCC on clinical outcomes—overall inpatient days of antibiotic therapy, test utilization, length of stay, and mortality—was determined via a multivariable regression model.

**Results:**

An overall 22 927 patient admissions were evaluated: 650 (4.1%) and 339 (4.8%) with BCC and 11 437 (71.8%) and 4648 (66.3%) with negative BC results from the Netherlands and the United States, respectively. Dutch and US patients with BCC had a mean ± SE 1.74 ± 0.27 (*P* < .001) and 1.58 ± 0.45 (*P* < .001) more days of antibiotic therapy than patients with negative BC results. They also had 0.6 ± 0.1 (*P* < .001) more BCs drawn. Dutch but not US patients with BCC had longer hospital stays (3.36 days; *P* < .001). There was no difference in mortality between groups in either cohort. AU remained higher in US but not Dutch patients with BCC in a subanalysis limited to BC obtained within the first 24 hours of admission.

**Conclusions:**

BCC remains associated with higher inpatient AU and health care utilization as compared with patients with negative BC results, although the impact on these outcomes differs by country.

## Teaser

The impact of blood culture contamination in clinical care was evaluated in 22 927 hospitalized adults with negative and contaminated blood cultures from the Netherlands and United States. Blood culture contamination was associated with unnecessary antibiotic administration and ancillary test use.

Blood cultures (BCs) are among the most frequently ordered microbiologic diagnostic tests in hospitals [1]. While BCs are essential for the optimal diagnosis and management of certain infections, the majority of BCs in routine clinical practice do not grow organisms [1], and of those that are positive, up to 40% to 55% represent BC contamination (BCC) [2–4]. BCC has become a major focus of antimicrobial stewardship because early studies found that antibiotics are given unnecessarily in up to 50% of patients with BCC due to BCC. Antibiotics are typically given to these patients for a week, increasing their risk of adverse events and developing antibiotic-resistant infections [5, 6]. Additionally, BCC is associated with additional resource use (eg, microbiological and radiologic testing), prolonged hospitalization, and increased health care costs in the United States and Europe [7, 8]. Many of these single-center studies are old and thus do not reflect the use of modern laboratory approaches to facilitate earlier identification of organisms growing in BC or the influence of the dissemination of diagnostic and antibiotic stewardship practices among health care workers. More recent studies have shown that implementation of rapid diagnostic tests for bacteremia coupled with antimicrobial stewardship support significantly reduces antibiotic use and health care resource utilization associated with BCC [9, 10].

To better understand the current impact of BCC on patients, we evaluated the impact of BCC on antibiotic use and health care utilization among hospitalized adults in the United States and the Netherlands.

## METHODS

### Study Design, Setting, and Population

We conducted a retrospective observational cohort study of adult patients who had at least 2 BCs performed at the Amsterdam University Medical Centers (AUMC) and The Johns Hopkins Medicine Health System (JHMHS) to compare clinical outcomes between patients with negative BC results and those with BCC. AUMC includes an academic hospital with 2 sites of 733 and 1002 beds in Amsterdam, the Netherlands. JHMHS includes 5 acute care hospitals in the Baltimore–Washington, DC region: a 1056-bed tertiary care teaching hospital, a 350-bed teaching hospital, and 3 community hospitals (264, 222, and 318 beds).

Inclusion criteria were patients ≥18 years of age who received at least 2 BCs during their hospitalization between 1 January 2016 and 31 December 2019 (AUMC) and from 1 January 2019 to 31 December 2019 (JHMHS). When a patient presented multiple times within the study period, each encounter was considered a unique visit. Exclusion criteria included patients who had only 1 BC set obtained during their hospitalization, were not admitted to the hospital, died within 48 hours of admission, or had an absolute neutrophil count ≤500 cells/µL on admission.

The AUMC and JHMHS institutional review boards waived the review of this study, as the Medical Research Involving Human Subjects Act did not apply.

### BC Collection and Processing

The health systems follow similar BC collection practices for bacteremia detection (2 sets of BC, 8–10 mL of blood per bottle in adults). Phlebotomy staff collect the majority of BCs in non–intensive care units (non-ICUs), while ICU BCs are usually collected by nursing and less commonly by physicians. BC processing included organism detection with either the BD BACTEC FX BCx system (Becton Dickinson) or the BacT/Alert 3D system (BioMerieux) at AUMC and the BD BACTEC FX BCx system at JHMHS. AUMC and JHMHS used matrix-assisted laser desorption ionization–time of flight mass spectrometry for microorganism identification (once grown in solid media). Additionally, JHMS used the Verigene BCID-GP panel. Both hospital systems alert their primary teams when Gram stains indicate positive BC results. There is no additional interpretation of results by the microbiology laboratory at JHMHS, while discussion of results may occur at AUMC. At AUMC and JHMHS, a “possible contaminant” comment appears for a single BC with commensal bacteria with an option to request susceptibility testing if needed.

### Antibiotic Stewardship Activities

All hospitals in AUMC and JHMHS have antibiotic stewardship programs, although they differ in their type of interventions and resources. For example, while both health systems perform daily postprescription review with feedback of intravenous (IV) vancomycin and broad-spectrum antibiotics, prior authorization for vancomycin is required in 2 of the 5 JHMHS hospitals (although it can be dispensed without approval in certain circumstances) and none of the AUMC sites.

### Data Collection and Definitions

Data were bulk extracted from the electronic health record system, deidentified, and analyzed by local investigators at AUMC and JHMHS separately.

Unique patient admissions were classified as true positive, BCC, or negative (Supplementary Figure 1). BC-negative cases were patients whose BCs were all negative during their hospitalization. A BC was considered a contaminant if ≥1 commensal organisms were identified in only 1 of a series of BCs collected within a 24-hour period. The full list of bacteria included as contaminants is shown in Supplementary Table 1. When these organisms were found in >1 BC set of a series collected within 24 hours, BCs were classified as true positive. Comorbidities were defined with the Charlson Comorbidity Index (CCI).

### Outcomes and Statistical Analysis

The primary outcome was overall inpatient antibiotic days of therapy. Secondary outcomes included overall inpatient IV vancomycin days of therapy, additional BC testing, imaging utilization, peripheral IV insertions, length of stay, and mortality. Primary and secondary outcomes were compared by linear regression for continuous outcomes and logistic regression for binary outcomes after adjusting for age, sex, CCI, and ICU length of stay between patients with BCC and those with negative BC results. We performed a subanalysis measuring outcomes within 14 days after BC collection at the BC level and a subanalysis of outcomes limited to BC obtained in first 24 hours of hospitalization, assuming that these antibiotics are more likely to be related to BC results [11]. Where appropriate, Wilcoxon rank sum and *t* test were used to compare medians and means, respectively.

## RESULTS

### Cohort

**Figure 1.**
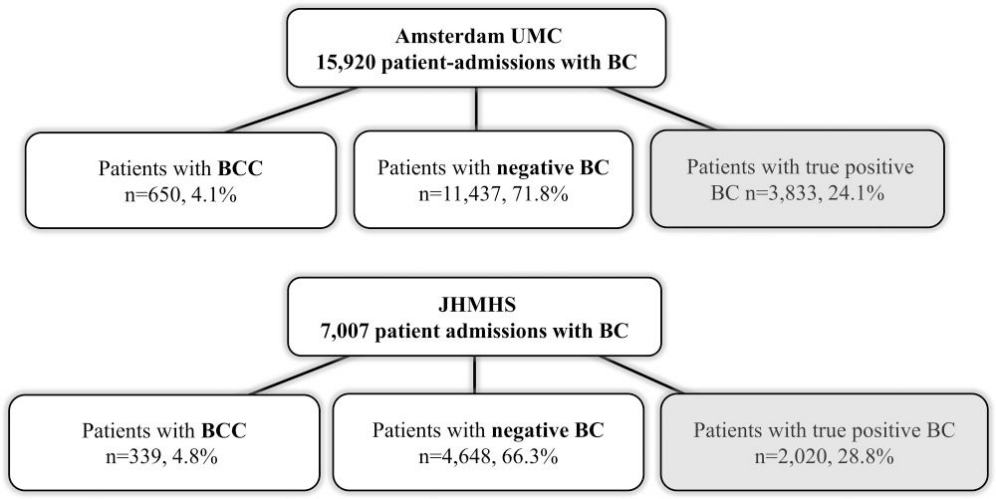
Number of patients evaluated and results. Analysis was performed in patients with negative blood cultures and those with blood culture contamination (ie, patients with true positive blood cultures were not included in the analysis). Amsterdam UMC, Amsterdam University Medical Centers; BC, blood culture; BCC, blood culture contamination; JHMHS, The Johns Hopkins Medicine Health System.

In total, 15 920 and 7007 patient admissions with BC at AUMC and JHMHS were analyzed. Among these, there were 650 (4.1%) and 339 (4.8%) with BCC and 11 437 (71.8%) and 4648 (66.3%) with negative BC results, respectively (Figure 1). The most common BC contaminants for each cohort are summarized in Supplementary Figure 2. Patient characteristics are described in Table 1.

**Table 1.** Baseline Characteristics of the Study Population Stratified by Health System and by BC Result

	AUMC Patients			JHMHS Patients		
Clinical Characteristic	With BCC (n = 650)	With Negative BC (n = 11 437)	Total (n = 12 087)	With BCC (n = 339)	With Negative BC (n = 4648)	Total (n = 4987)
Demographics						
Age, y, mean (SD)	61.9 (17.3)	58.5 (17.8)	58.5 (17.8)	63.8 (19.0)	60.0 (18.5)	60.2 (18.6)
Women	302 (46.5)	5222 (45.7)	5218 (43.2)	167 (49.3)	2179 (46.9)	2346 (47.0)
Body mass index, mean (SD)	26.7 (5.7)	25.7 (6.0)	25.7 (6.0)	29.2 (17.5)	27.7 (8.5)	27.8 (9.3)
Comorbidities						
History of malignancy, organ transplantation, or AIDS	62 (9.5)	1184 (10.4)	1246 (10.3)	107 (31.6)	2160 (46.5)	2267 (45.5)
Diabetes	37 (5.7)	495 (4.3)	532 (4.4)	112 (33.0)	1611 (34.7)	1723 (34.6)
Chronic kidney disease	22 (3.4)	257 (2.2)	279 (2.3)	14 (4.1)	138 (3.0)	152 (3.0)
Pacemaker	3 (0.5)	81 (0.7)	84 (0.7)	94 (27.7)	1045 (22.5)	1139 (22.8)
CCI, median (IQR)	2 (1–4)	2 (1–3)	2 (1–3)	7 (4–9)	7 (4–9)	7 (4–9)
Most common infection-related admitting diagnoses						
Sepsis	6 (0.9)	72 (0.6)	78 (0.6)	43 (12.7)	414 (8.9)	457 (9.2)
Pneumonia	54 (8.3)	778 (6.8)	832 (6.9)	30 (8.8)	440 (9.5)	470 (9.4)
Skin and soft tissue infections	5 (0.8)	200 (1.7)	205 (1.7)	21 (6.2)	254 (5.5)	275 (5.5)
Urinary tract infections	19 (2.9)	463 (4.0)	482 (4.0)	12 (3.5)	60 (1.3)	72 (1.4)
Biliary infections	4 (0.6)	359 (3.1)	363 (3.0)	4 (1.2)	68 (1.5)	72 (1.4)
ICU admissions	211 (32.5)	2093 (18.3)	2304 (19.1)	114 (33.6)	1665 (35.8)	1779 (35.7)

Data are presented as No. (%) unless noted otherwise.

Abbreviations: AUMC, Amsterdam University Medical Centers; BC, blood culture; BCC, blood culture contamination; CCI, Charlson Comorbidity Index; ICU, intensive care unit; JHMHS, The Johns Hopkins Medicine Health System.

### Impact of BCC

After adjusting for age, sex, CCI, and ICU length of stay, patients with BCC received significantly more days of antibiotics than those with negative BC results (mean, 1.74 and 1.58 additional days of antibiotics for Dutch and US cases, respectively; *P* < .001 for both; Table 2; for results of bivariate analysis, see Supplementary Table 2). IV vancomycin utilization was higher in US patients with BCC as compared with patients with negative BC results (*P* < .001); no significant difference was seen in the Dutch cohort. Additionally, BCC was associated with higher use of BC for both cohorts. While more imaging studies were obtained in AUMC patients with BCC (1.36 more images, *P* < .001), more imaging studies were obtained in US patients without BCC (3.08 fewer images among cases, *P* = .007). There was more documented use of peripheral lines (0.37 insertions, *P* < .001) and a 3-day increase in hospital length of stay (*P* < .001) for Dutch patients with BCC vs those without. There were no differences in in-hospital or 30-day mortality between patients with and without BCC for either cohort. There were no significant differences among hospitals within the same health system.

**Table 2.** Multivariate Analysis (Linear and Logistic Regression): Clinical Outcomes in Patients With Blood Culture Contamination vs Negative Blood Culture Results

	Adjusted Analysis ^a^			
Outcome	AUMC	*P* Value ^b^	JHMHS	*P* Value ^b^
β (SE)				
Antibiotics, d	1.74 (0.27)	**<**.**001**	1.58 (0.45)	**<**.**001**
IV vancomycin, d	0.037 (0.03)	.187	1.36 (0.24)	**<**.**001**
No. of blood cultures	0.60 (0.08)	**<**.**001**	0.68 (0.10)	**<**.**001**
No. of images	1.36 (0.18)	**<**.**001**	–3.08 (1.14)	.**007**
No. of peripheral IV insertions	0.37 (0.06)	**<**.**001**	0.008 (0.023)	.725
Length of stay, d	3.36 (0.59)	**<**.**001**	–0.96 (0.86)	.265
Odds ratio (95% CI)				
In-hospital mortality	1.21 (0.88–1.61)	.216	0.85 (0.57–1.29)	.457
30-d mortality	0.78 (0.54–1.10)	.172	0.48 (0.21–1.09)	.079

Abbreviations: AUMC, Amsterdam University Medical Centers; IV, intravenous; JHMHS, The Johns Hopkins Medicine Health System.

^a^Adjusted for age, sex, Charlson Comorbidity Index, and ICU length of stay.

^b^Bold indicates *P* < .05.

A subanalysis of outcomes evaluated 14 days after BC collection revealed similar results (Table 3). Similarly, a subanalysis limited to results of BC collected within the first 24 hours of hospitalization showed higher inpatient antibiotic use among patients with BCC in the United States but not in the Dutch cohort (Table 4).

**Table 3.** Subanalysis: Clinical Outcomes Associated With BCC and Negative BC

	AUMC			JHMHS		
Clinical Characteristic	BCC (n = 2004)	Negative BC (n = 35 327)	Total (n = 37 331)	BCC (n = 1158)	Negative BC (n = 13 077)	Total (14 235)
DOT						
After BC ^a^	4.72 (4.79)	4.34 (4.51)	4.36 (4.52)	4.99 (5.03)	3.94 (4.90)	4.03 (4.92)
Before BC	2.71 (4.58)	2.10 (3.73)	2.13 (3.78)	3.77 (5.45)	3.54 (7.95)	3.56 (7.78)
DOT: IV vancomycin						
After BC ^a^	0.16 (1.14)	0.07 (0.69)	0.07 (0.72)	2.50 (3.01)	1.58 (2.76)	1.66 (2.79)
Before BC	0.04 (0.54)	0.03 (0.44)	0.03 (0.45)	1.70 (2.76)	1.47 (4.15)	1.49 (4.06)
No. of images	10.00 (10.89)	7.58 (8.31)	7.71 (8.48)	8.90 (12.38)	10.11 (12.90)	10.01 (12.86)
No. of peripheral IV insertions	1.30 (1.42)	0.95 (1.17)	0.97 (1.19)	0.19 (0.40)	0.16 (0.36)	0.16 (0.37)

Data are presented as mean (SD). Analysis was performed according to the BC result as opposed to the patient level.

Abbreviations: AUMC, Amsterdam University Medical Centers; BC, blood culture; BCC, blood culture contamination; DOT, days of antibiotic therapy; IV, intravenous; JHMHS, The Johns Hopkins Medicine Health System.

^a^“After BC” refers to events occurring within 14 days after BC collection.

**Table 4.** Multivariate Analysis: Outcomes of Patients With BCC vs Negative BC

	Adjusted Analysis, ^a^ β (SE)			
Outcome	AUMC	*P* Value ^b^	JHMHS	*P* Value ^b^
Antibiotics, d	−0.068 (0.18)	.708	6.000 (0.107)	**<**.**001**
IV vancomycin, d	0.028 (0.02)	.204	3.260 (0.001)	**<**.**001**
No. of BC	−0.016 (0.04)	.700	0.512 (0.112)	**<**.**001**
No. of images	0.842 (0.13)	**<**.**001**	4.866 (0.142)	**<**.**001**
No. of peripheral IV insertions	0.409 (0.14)	.**003**	0.189 (0.009)	**<**.**001**

Data are based on results of BC collected in the first 24 hours of admission. AUMC, n = 9069 (BCC, n = 422; negative BC, n = 8647). JHMHS, n = 2033 (BCC, n = 127; negative BC, n = 1906).

Abbreviations: AUMC, Amsterdam University Medical Centers; BC, blood culture; BCC, blood culture contamination; IV, intravenous; JHMHS, The Johns Hopkins Medicine Health System.

^a^Adjusted for age, sex, Charlson Comorbidity Index, and intensive care unit length of stay.

^b^Bold indicates *P* < .05.

## DISCUSSION

The impact of BCC on inpatient antibiotic use was studied in 2 health systems, including 2 hospitals in the Netherlands and 5 hospitals in the United States. We found that patients with BCC had higher overall inpatient antibiotic use and health care resource utilization as compared with patients with negative BC results after adjusting for age, sex, CCI, and ICU length of stay, although clinical consequences differed by country. While antibiotic use remained higher in patients with BCC vs patients with negative BC results on the main and sensitivity analyses for the US cohort, this was not the case for the Dutch cohort. It is possible that the main analysis did not capture all the confounders and overestimated antibiotic use among Dutch patients. Other clinical consequences, such as higher utilization of images and peripheral IV insertions, remained similar between the main and sensitivity analyses in the Dutch cohort.

Previous studies showed that patients with BCC received approximately 5 to 7 additional days of antibiotics than patients with negative BC results [5]. Studies have shown that implementation of rapid diagnostic testing for bacteremia coupled with review of positive BC by antibiotic stewardship programs may lead to fewer patients exposed and shorter exposure to unnecessary antibiotics of patients with BCC [9, 10], although a recent study rebutted this [11]. Our study consisted of a mix of large and medium-size hospitals that have implemented different strategies to help mitigate inappropriate antibiotic use related to BCC: (1) comments in the electronic health record system indicating when a result is likely a contaminant; (2) prior approval by the antimicrobial stewardship team before pharmacy dispensing; (3) postprescription review and feedback of selected antibiotics, including IV vancomycin and broad-spectrum antibiotics; and (4) rapid molecular testing for bacteremia detection, which provides early knowledge of bacterial species and resistance genes. Despite such interventions, patients with BCC had higher overall inpatient antibiotic utilization as compared with patients with negative BC results after adjusting for clinical characteristics, including comorbidities and ICU length of stay. Higher use of IV vancomycin in US patients but not Dutch patients with BCC is likely related to antimicrobial susceptibility patterns between the countries, as published in the literature [12].

Inclusion of data from 2 countries increases generalizability of our results. Initiatives to reduce unwanted consequences related to BCC may include steps to reduce contamination at the time of BC collection, decrease unnecessary BC orders, and improve interpretation of BC results and need for treatment by clinicians [2, 13]. Data show that most BCs ordered in hospitalized patients are negative [1], and the yield of BC is significantly reduced when obtained after 24 hours of hospitalization, highlighting the need to improve BC-ordering practices [3]. Our group has shown that approximately 30% of BCs can be reduced safely in the emergency department setting in the Netherlands through the use of a prediction tool that uses easily available electronic medical record data [14], as well as in the inpatient setting in the United States through the use of an algorithm with indications on when to draw BCs based on the probability of bacteremia according to the presenting signs and symptoms [15, 16]. Moreover, while BCC has received renewed attention with a new recommended threshold by the Clinical and Laboratory Standards Institute (BCC <1%) [17], attention to other BC quality indicators, such as single sets, is needed. A recent evaluation of national BC practices in Israel showed that 45% to 94% of all BCs obtained from hospitalized patients were single sets [18]. A survey of BC practices among clinicians in the United States revealed that clinicians were frequently unaware of the adequate number of BC sets needed to detect bacteremia [19]. These data highlight the importance of tracking BC quality indicators beyond BCC and working with relevant stakeholders on improving BC utilization and performance.

Our study has some limitations. There are several factors that might cause underestimation of the impact of BCC in this study. First, we excluded patients with only 1 BC set drawn during their hospitalization. Second, BCs that grew a skin contaminant in >1 BC in 24 hours were considered true positives when many of these may have represented BCC. Additionally, for patients who had multiple BC series during their hospitalization, if 1 of these included only 1 BC set and if this single BC grew a contaminant, it was classified as true positive, as manual chart review was not feasible to perform a more accurate adjudication. Third, we included only inpatient antibiotic use because outpatient antibiotic use data were not available for all patients. Fourth, the US and Dutch cohorts differed in comorbidities and admitting diagnoses—for example, more patients in the United States had a history of diabetes and pacemaker implantation, and more US patients were admitted for sepsis. It remains uncertain whether these are true differences in patient population between the countries vs differences in documentation and coding by clinicians; for instance, there is a big emphasis on documenting compliance with sepsis bundles in US hospitals. However, this should not affect interpretation of results, as the clinical outcomes were assessed between BCC and negative BC results within each cohort. Finally, we did not capture indications for antibiotic or health care resource utilization; therefore, we cannot ascertain causality. Yet, our results are consistent with prior and more recent studies addressing potential consequences of BCC.

In summary, our findings show that while the impact of BCC on patients may have lessened in recent years, it remains an important source of unnecessary antibiotic use and health care resource use in hospitalized patients in the United States and the Netherlands.

## Supplementary Material

ofad644_Supplementary_Data

## Supplementary Data

Supplementary materials are available at *Open Forum Infectious Diseases* online. Consisting of data provided by the authors to benefit the reader, the posted materials are not copyedited and are the sole responsibility of the authors, so questions or comments should be addressed to the corresponding author.

## Notes

***Financial support***. This work was supported in part by the US Centers for Disease Control and Prevention Epicenters Program (1 U54 CK000617-01).
