# Supplementary material for: Impact of Blood Culture Contamination on Antibiotic Use, Resource Utilization, and Clinical Outcomes: A Retrospective Cohort Study in Dutch and US Hospitals
Source: Open Forum Infect Dis. 2023 Dec 22;11(2):ofad644. doi: 10.1093/ofid/ofad644 (PMC10836193; doi:10.1093/ofid/ofad644)

**Supplementary Material**

**Content**

1. **Suppl. Figure 1**: Approach to classification of blood culture results. Page 2.
2. **Suppl. Table 1.** List of microorganisms that were considered contaminants if the blood culture contamination definition was met (i.e., not more than once per 24-hour period out of multiple blood cultures). Pages 3-4.
3. **Suppl. Table 2.** Unadjusted overall clinical outcomes associated with blood culture contamination. Page 5.
4. **Suppl. Figure 2**: Most common blood culture contaminants found in both cohorts. Page 6.

**Suppl. Figure 1:** Approach to classifying blood culture (BC) results.

**Suppl. Table 1.** List of microorganisms that were considered contaminants if the blood culture contamination definition was met (i.e., not more than once per 24-hour period out of multiple blood cultures).

| *Bacillus spp.* (except *B. anthracis or B. cereus/thuringiensis*) |
| --- |
| Staphylococci (other than *S. aureus/S. lugdunensis/S. saprophyticus/S. pettenkoferi*) |
| *Corynebacterium spp.* (except *C. diphtheriae*) |
| *Propionibacterium spp.* |
| *Aerococcus spp.* |
| *Micrococcus spp.* |
| *Abiotropia spp.* |
| *Granulicatella spp.* |
| Viridans group streptococci |
| **Additional/renamed organisms considered contaminants** |
| *Niallia (Bacillus) circulans* |
| *Priestia (Bacillus) megaterium* |
| *Ureibacillus (Lysinibacillus) sassiliensis* |
| *Paenibacillus glucanolyticus* |
| *Paenibacillus vulneris* |
| *Paenibacillus urinalis* |
| *Paenibacillus validus* |
| *Paenibacillus polymyxa* |
| *Paenibacillus campinasensis* |
| *Paenibacillus alvei* |
| *Paenibacillus azotofixans* |
| *Paenibacillus maceran* |
| *Paenibacillus spp.* |
| *Paenibacillus humicus* |
| *Paenibacillus lautus* |
| *Paenibacillus odorifer* |
| *Paenibacillus provencensis* |
| *Paenibacillus timonensis* |
| *Paenibacillus dakarensis* |
| *Paenibacillus massiliensis* |
| *Rothia dentocariosa* |
| *Rothia amarae* |
| *Rothia aeria* |
| *Rothia mucilanginosa* |
| *Rothia terrae* |
| *Kocuria kristinae* |
| *Kocuria palustris* |
| *Kocuria rhizophila* |
| *Kocuria rhizophila group* |
| *Kocuria rosea* |
| *Kocuria varians* |
| *Dermabacter hominis* |
| *Dermabacter jinjuensis* |
| *Brevibacterium casei* |
| *Brevibacterium epidermidis* |
| *Brevibacterium iodinum* |
| *Brevibacterium linens* |
| *Brevibacterium luteolum* |
| *Brevibacterium mcbrellneri* |
| *Brevibacterium paucivoans* |
| *Brevibacterium ravenspurgense* |
| *Brevibacterium sanguinis* |

**Suppl. Table 2.** Unadjusted overall clinical outcomes associated with blood culture contamination (BCC).

| **Outcome** | **AUMC**  **Patients with BCC**  **(n=650)** | **AUMC Patients with negative BC**  **(n=11,437)** | **AUMC *P*-value** | **JHMHS Patients with BCC**  **(n=339)** | **JHMHS Patients with negative BC**  **(n=4,648)** | **JHMHS *P*-value** |
| --- | --- | --- | --- | --- | --- | --- |
| Days of antibiotics, median (IQR) | 4 (1-9) | 3 (0-6) | **<0.001** | 6 (3-11) | 4 (1-9) | **<0.001** |
| Days of IV vancomycin, median (IQR) | 0 (0-0) | 0 (0-0) | **0.003** | 3 (1-5) | 1 (0-3) | **<0.001** |
| Blood culture sets, median (IQR) | 2 (1-3) | 2 (1-2) | **<0.001** | 3 (2-4) | 2 (2-3) | **<0.001** |
| Images, median (IQR) | 4 (2-9) | 3 (1-5) | **<0.001** | 10 (5-24) | 12 (6-26) | **0.026** |
| Peripheral IV insertions, median (IQR) | 1 (1-3) | 1 (0-2) | **<0.001** | 0 (0-0) | 0 (0-0) | 0.741 |
| Length of stay, median (IQR) | 9.8 (4.1-24.7) | 6.8 (3.3-13.9) | **<0.001** | 8 (5-16) | 10 (5-19) | 0.122 |
| In-hospital mortality % | 9.1% | 5.6% | **<0.001** | 8.3% | 9.3% | 0.509 |
| 30-day mortality %* | 1.5% | 2.5% | **0.166** | 1.9% | 3.9% | 0.076 |

IQR: interquartile range, IV: intravenous. *Excluding those who died within the hospital.

**Suppl. Figure 2:** Counts of the most commonly found blood culture contaminants in both cohorts. JHMS: Johns Hopkins Medicine Health System, AUMC: Amsterdam University Medical Center.


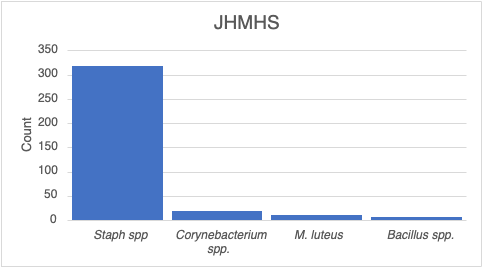

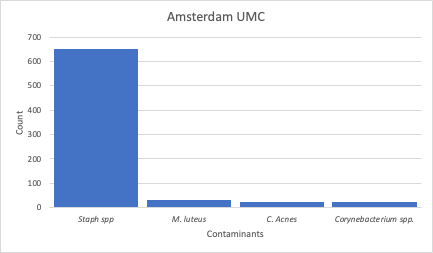

Supplement: ofad644_Supplementary_Data [file ofad644_supplementary_data.docx]
